# Near-Infrared-Induced Hydrophobic Characteristic of Black TiO_2_ Coatings Showing Antibacterial and Immunomodulatory Properties

**DOI:** 10.3390/jfb17090432

**Published:** 2026-08-28

**Authors:** Yulin Gao, Kai Li, Qiang Chen, Pingtuo Wang, Aoshuang Xun, Yi Ding, Heng Ji, Xuebin Zheng

**Affiliations:** 1 Key Laboratory of Inorganic Coating Materials CAS, Shanghai Institute of Ceramics, Chinese Academy of Sciences, 1295 Dingxi Road, Shanghai 200050, China; gaoyulin24@mails.ucas.ac.cn (Y.G.); 232223058@st.usst.edu.cn (Q.C.); 22021901019@mail.hnust.edu.cn (P.W.); xunaoshuang23@mails.ucas.ac.cn (A.X.);; 2 Center of Materials Science and Optoelectronics Engineering, University of Chinese Academy of Sciences, 19 Yuquan Road, Beijing 100049, China

**Keywords:** black TiO_2_ coating, atmospheric plasma spray, NIR responsiveness, surface wettability, orthopedic Ti implant

## Abstract

Surface wettability is a critical factor influencing the biological performance of orthopedic Ti implants. The native TiO_2_ film on Ti undergoes changes in wettability under ultraviolet (UV) irradiation. However, the limited tissue penetration of UV light compared with near-infrared (NIR) light restricts its potential clinical application. In this study, a black TiO_2_ (b-TiO_2_) coating with NIR-responsive wettability was fabricated directly on a Ti substrate using a one-step atmospheric plasma spraying process. Under 808 nm NIR irradiation, the water contact angle of the b-TiO_2_ coating increased from 0° to 154.4 ± 4.0°, indicating a transition from a superhydrophilic to a stable superhydrophobic state. FTIR and XPS analyses showed that NIR-induced photothermal heating promoted the removal of surface hydroxyl groups and the passivation of oxygen-deficient sites, thereby driving the wettability transition. Among TiO_2_ coatings with hydrophilic, intermediate-wettability, and hydrophobic surfaces, the hydrophobic coating effectively directed macrophage polarization toward the anti-inflammatory M2 phenotype and delivered slightly superior osteoblast activity. It also markedly inhibited *Staphylococcus aureus* adhesion, achieving an anti-adhesion efficiency of 98.61%. These findings demonstrate that NIR irradiation can regulate the wettability of plasma-sprayed b-TiO_2_ coatings and provide concurrent immunomodulatory and antibacterial effects. This approach may support the development of light-responsive surfaces for orthopedic implants.

## 1. Introduction

Ti and its alloys are commonly used bone implant materials due to their excellent biocompatibility [1,2], which is mainly attributed to the formation of a dense TiO_2_ film on the surface [3]. However, the intrinsic bioinertness of TiO_2_ often impedes effective implant osseointegration [4,5,6]. Meanwhile, bacterial infection represents another major risk factor impeding the osseointegration process [7,8]. Surface wettability is one of the most critical physicochemical properties governing bacterial adhesion and mammalian cell behaviors on implants [9,10]. Appropriate surface wettability significantly influences osteoblast adhesion and differentiation [4,11], regulates macrophage function [12,13,14,15], and exerts an inhibitory effect on bacterial adhesion [16], thereby promoting osseointegration [9,17]. Therefore, manipulating the surface wettability of TiO_2_ coatings is of great significance for enhancing osteogenic and antibacterial functionalities.

Recent studies have increasingly focused on diverse strategies for tailoring the surface wettability of TiO_2_ coatings, including the regulation of surface roughness and chemistry, modulation of crystal structure, and application of ultraviolet (UV) irradiation. In a study by Jaggessar et al. [18], hydrophilic nano-TiO_2_ coatings were prepared via a hydrothermal method. It was found that the coating surface with a contact angle between 35° and 50° exhibited the highest level of osteoblast activity. Lv et al. [19] combined atomic layer deposition (ALD) with self-assembled monolayers to fabricate TiO_2_ films with hydrophilic–hydrophobic stripe patterns. They demonstrated that the surface wettability of TiO_2_ films can be utilized to control the immune response by steering macrophage polarization. Liu and colleagues [20] prepared TiO_2_ thin films with different crystal structures (amorphous and anatase phases) via ALD at different temperatures. The amorphous TiO_2_ coating exhibited a larger water contact angle and better antibacterial performance. A study by Shayan et al. [21] reported that UV irradiation increased the surface hydrophilicity of PMMA/TiO_2_ nanocomposites, which promoted the adhesion and proliferation of osteoblasts. Although the above-mentioned TiO_2_ films can respond to UV light along with a wettability change, the limited tissue penetration depth of UV light restricts its clinical application.

Black TiO_2_ (b-TiO_2_) rich in oxygen vacancies exhibits a narrower bandgap which extends its light adsorption from UV light to NIR light [22]. Laser irradiation, chemical reduction, and plasma treatment are common methods for fabricating b-TiO_2_ powders and coatings [23,24]. In order to endow b-TiO_2_ with a hydrophobic characteristic, post-treatment strategies such as dark storage and low-temperature annealing are required [25,26,27]. Hu et al. achieved a hydrophobic CeO_2_/b-TiO_2_ coating by storing it in darkness for 3 days [25]. Pan et al. [26] fabricated PFOS-modified TiO_2_ coatings, which enabled hydrophobic–hydrophilic conversion following low-temperature annealing and UV light irradiation. Similarly, Jiao et al. [27] prepared multiscale TiO_2_ arrays using femtosecond laser ablation, which realized reversible wettability regulation through UV irradiation and low-temperature annealing. Nevertheless, existing wettability regulation strategies for b-TiO_2_ coatings still exhibit obvious limitations. For example, dark storage is time-consuming, and low-temperature annealing requires specialized equipment. These make it difficult to achieve the rapid regulation of surface wettability. Considering the NIR-induced heat conversion characteristic of b-TiO_2_, previous studies have used it as a photothermal agent for bone tumor treatment. The NIR-induced heat conversion in b-TiO_2_ may promote the removal of surface hydroxyl groups in the atmosphere, which plays a role in surface wettability.

Atmospheric plasma spray (APS) is widely used to fabricate coatings because of its high plasma jet temperature and rapid deposition rate. Moreover, rapid heating during the spraying process fully melts TiO_2_ particles, while subsequent rapid cooling promotes oxygen loss, leading to the formation of oxygen vacancies and Ti^3+^ defects [28]. This unique thermal history makes APS a suitable technique for preparing b-TiO_2_ coatings. Accordingly, in this study, b-TiO_2_ coatings were fabricated directly on Ti substrates via a one-step APS process. White TiO_2_ (w-TiO_2_) coatings, obtained by annealing the b-TiO_2_ coatings, were used as controls. The dynamic change in the surface wettability of b-TiO_2_ under NIR light irradiation was investigated. The underlying mechanism of the NIR-induced hydrophobic characteristic was clarified. Furthermore, the effects of surface wettability on the antibacterial, immunomodulatory, and osteogenic properties of the coatings were evaluated, including the inhibition of *Staphylococcus aureus* (*S. aureus*), modulation of macrophage morphology, and promotion of osteoblast activity.

## 2. Materials and Methods

### 2.1. Coating Preparation

Ti alloy disks (Ti-6Al-4V, Ø10 mm × 1 mm; Dongwan Baotai Industrial Co., Dongwan, China) were used as substrates. Black titanium dioxide (b-TiO_2_; Shandong Zibo Linkai Chemical Materials Co., Ltd., Zibo, China) and white titanium dioxide (w-TiO_2_; same supplier) coatings were deposited on the substrates by atmospheric plasma spraying (APS, F4-VB torch; Oerlikon Metco AG, Wohlen, Switzerland). Notably, the as-sprayed w-TiO_2_ coatings initially exhibited a black appearance, which transitioned to white after annealing in air at 600 °C for 2 h, indicating the formation of an oxygen-vacancy-rich (sub-stoichiometric) phase during spraying that was subsequently re-oxidized upon thermal treatment. All coated samples were ultrasonically cleaned in absolute ethanol for 10 min and dried at 50 °C prior to further characterization or use.

### 2.2. Surface Characterization

Surface morphology and elemental composition were examined by field-emission scanning electron microscopy (FE-SEM, Hitachi Ltd., Tokyo, Japan) coupled with an energy-dispersive X-ray spectrometer (EDS). The crystalline phase was identified by X-ray diffraction (XRD, D/max-2550, Rigaku Corporation, Akishima, Japan). Raman and FTIR spectra were obtained by a Raman spectrometer (Raman, LabRAM HR Evolution, Horiba, Japan) and an FTIR spectrometer (FTIR, Nicolet S50, Thermo Scientific, Waltham, MA, USA), respectively. The surface chemical state of Ti and O was analyzed by X-ray photoelectron spectroscopy (XPS, EscaLab 250, Thermo Fisher Scientific, Waltham, MA, USA), and spectra were deconvoluted using Avantage (Version 6.9). Static water contact angles were measured by the sessile drop method (FCA2000A, Aifes, Shanghai, China). Photothermal response was recorded by irradiating the coatings with an 808 nm NIR laser (Beijing Blueprint Technology Co., Ltd., Beijing, China) at a power density of 2 W/cm^2^ while simultaneously capturing real-time thermal images with an infrared thermal imaging system (Hangzhou Hikmicro Sensing Technology Co., Ltd., Hangzhou, China).

### 2.3. Anti-Adhesion Effect Against Staphylococcus aureus

*S. aureus* was used as the model strain. A single colony was inoculated into 5 mL of Luria–Bertani (LB) medium broth and cultured overnight at 37 °C with shaking. The overnight culture was diluted 1:100 in 70 mL of fresh LB broth and grown under the same conditions to the mid-log phase. Bacteria were collected by centrifugation, washed twice with phosphate-buffered saline (PBS), and resuspended in PBS to a final concentration of 10^7^ CFU/mL^−1^. Three TiO_2_ coatings with different surface wettability states (hydrophobic, hydrophilic, and intermediate-wettable surface) were used. Each TiO_2_ sample (10 mm Ø × 1 mm) was placed in an individual well of a 24-well plate and immersed in 1 mL of the bacterial suspension. The plate was covered, sealed in a Petri dish to maintain humidity, and incubated statically at 37 °C for 24 h. After incubation, samples were divided into two subsets. Unwashed subset: The coated sample was transferred directly to 1 mL sterile PBS and ultrasonicated for 3 min to detach all adhered bacteria. PBS-washed subset: The coated sample was gently rinsed with 1 mL PBS to remove loosely attached bacteria, then transferred to 1 mL PBS and ultrasonicated identically. Suspensions were serially diluted in PBS, and 100 µL aliquots were spread on LB agar plates. Plates were incubated at 37 °C for 24 h, and colonies were counted. The antibacterial rate R was calculated as R = (T_0_ − T_1_)/T_0_ × 100%, where T_0_ and T_1_ are the mean colony counts (CFU mL^−1^) on the unwashed control and unwashed test samples, respectively.

### 2.4. Cell Culture

RAW264.7 cells were obtained from the Shanghai Cell Bank of the Chinese Academy of Sciences and maintained in Dulbecco’s modified Eagle’s medium (DMEM, Gibco, Waltham, MA, USA) supplemented with 10% newborn calf serum and 1% penicillin–streptomycin (Gibco). Cultures were incubated at 37 °C in an incubator. The culture media were refreshed every two days. The cells were detached by scraping when they reached 70–80% confluence.

The murine pre-osteoblastic cell line MC3T3-E1 was obtained from the Cell Bank of the Chinese Academy of Sciences and cultured in α-minimum essential medium (MEM; Hyclone, Logan, UT, USA) supplemented with 10% fetal bovine serum (FBS; Wisent, Nanjing, China) and 1% penicillin–streptomycin (Gibco). The cells were cultured at 37 °C in a humidified 5% CO_2_ atmosphere, with the medium replaced every 2 days.

### 2.5. Surface Marker Flow Cytometry

RAW264.7 cells were seeded on the sterilized TiO_2_ coatings in a 6-well plate at a density of 5 × 10^5^ cells per well. After 3 days, adherent and suspended cells were harvested by 0.05% EDTA in PBS, washed in PBS and counted. Subsequently, the cells were treated with antibodies specific for CCR7 (CCR7-APC, Bioscience, Carlsbad, CA, USA) and CD206 (CD206-PerCP-Cy5, Bioscience, Carlsbad, CA, USA). A flow cytometry instrument (BD Biosciences, Carlsbad, CA, USA) was employed to detect the markers of polarization. M1 macrophages were defined as CCR7^+^ CD206^−^ and M2 as CD206^+^ CCR7^−^, with the results expressed as the % of viable, single-cell events. Data were analyzed with FlowJo (Version 10.10.1) software (TreeStar, Ashland, OR, USA).

### 2.6. Immunofluorescence Staining of Macrophage Polarization Markers

RAW264.7 cells were seeded onto the sterilized TiO_2_-coated disks (ø10 mm × 2 mm) in 24-well plates at a density of 1 × 10^4^ cells per well. After 24 h of culture, the samples were washed with PBS, fixed with 4.0% paraformaldehyde, permeabilized with 0.2% Triton X-100, and blocked with 1.0% bovine serum albumin. Samples were then incubated overnight at 4 °C with primary antibodies against inducible nitric oxide synthase (iNOS; eBioscience, Thermo Fisher Scientific, Waltham, MA, USA) and arginase-1 (Arg-1; eBioscience, San Diego, CA, USA). After PBS washes, samples were incubated for 1 h at room temperature in the dark with fluorophore-conjugated secondary antibodies. Nuclei were counterstained with DAPI. After a final three PBS washes, samples were imaged on a confocal laser scanning microscope (TCS SP5 II, Leica, Microsystems GmbH, Wetzlar, Germany).

### 2.7. Cell Proliferation

MC3T3-E1 cells were seeded on TiO_2_-coated disks in a 24-well plate at a density of 5 × 10^4^ cells per well. Cell proliferation was measured for 7 and 14 days using CCK-8 (Tongren Institute of Chemistry, Kyushu Island, Japan) by reading OD_450_ on a Multiskan Sky microplate reader (Thermo Scientific).

### 2.8. Alkaline Phosphatase (ALP) Activity

MC3T3-E1 cells were seeded onto TiO_2_-coated disks in 24-well plates at 5 × 10^4^ cells/well and cultured in osteogenic differentiation medium. After 7 and 14 days, cells were lysed with 1% Triton X-100 and centrifuged at 12,000 rpm, and supernatants were collected. Alkaline phosphatase (ALP) activity and total protein were quantified by absorbance at 405 nm using a Multiskan Spectrum microplate reader (Thermo Fisher Scientific, Waltham, MA, USA). ALP activity was determined using p-nitrophenyl phosphate as the substrate, while total protein was measured via a standard protein assay. Total protein concentration was calculated according to the linear equation derived from a standard curve, and ALP activity was normalized to total protein content to account for variations in cell number.

### 2.9. ECM Mineralization

Cells were seeded at a density of 2 × 10^4^ cells per well onto each sample in 24-well plates, using osteogenic differentiation medium. After 14 and 21 days, cells were fixed with 4% paraformaldehyde and stained with alizarin red S solution for 0.5 h at 37 °C to visualize mineralized nodules. Then, the samples were thoroughly washed with PBS and incubated with 500 μL of 10% cetylpyridinium chloride (Sigma-Aldrich, St. Louis, MO, USA) for 15 min. A microplate reader was employed to quantify ECM mineralization by measuring the absorbance of the resulting solution at 590 nm.

### 2.10. Statistical Analysis

All data were expressed as the mean ± standard deviation (SD) of three parallel experiments (sample size *n* = 3). A one-way ANOVA and Sidak multiple comparison test were performed to analyze statistical differences. The degree of significance was indicated by * *p* < 0.05, ** *p* < 0.01, *** *p* < 0.001 or **** *p* < 0.0001.

## 3. Results and Discussion

### 3.1. Coating Characterization

**Figure 1:**
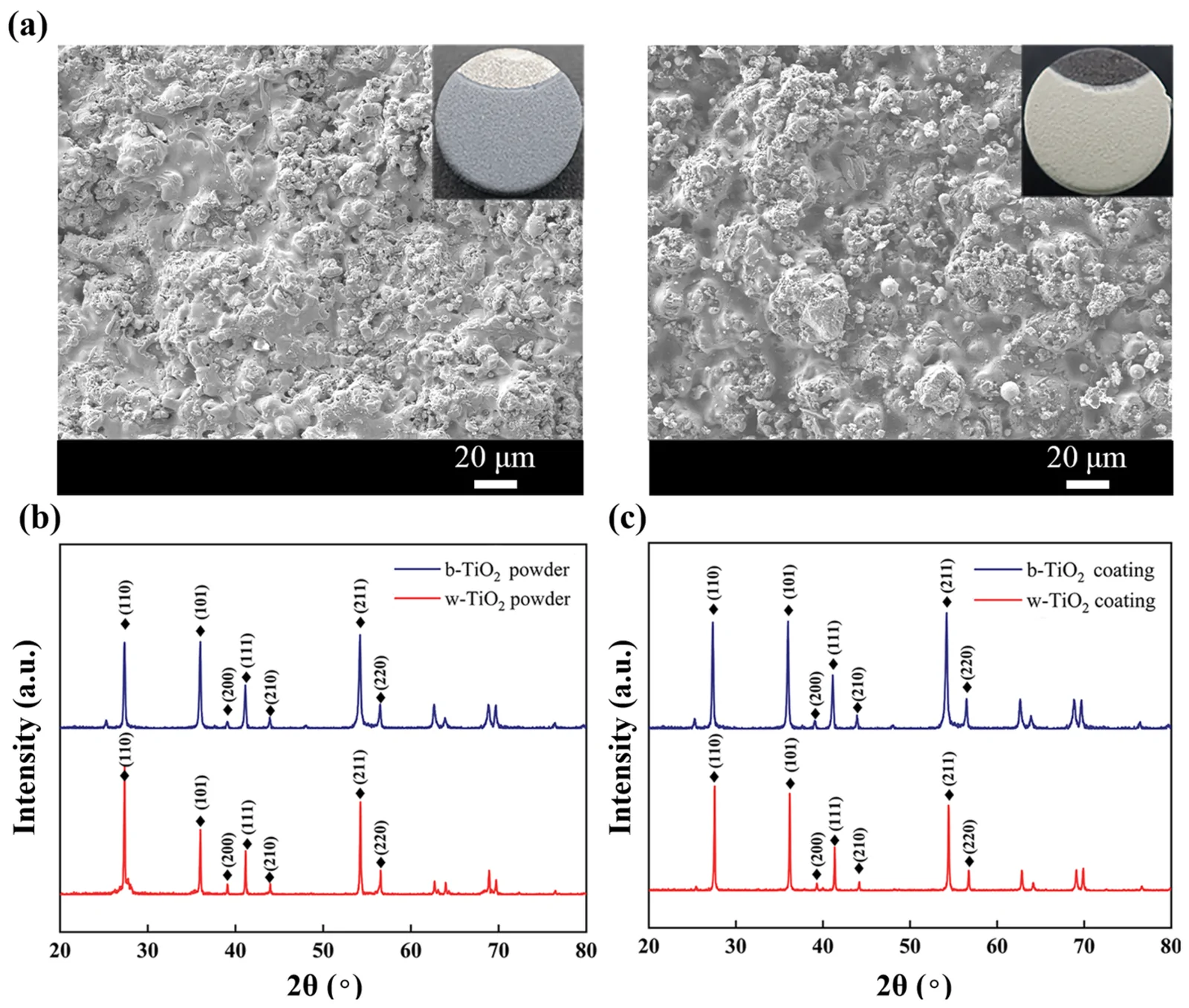
(**a**) SEM images of b-TiO_2_ and w-TiO_2_ coatings with optical photographs shown in upper-right insets. XRD patterns of (**b**) TiO_2_ powders and (**c**) corresponding TiO_2_ coatings.

Figure 1a shows the macroscopic photographs and surface micrographs of the b-TiO_2_ and w-TiO_2_ coatings. Both coatings exhibited the characteristic microstructure of plasma-sprayed coatings, comprising molten and semi-molten splats; such lamellar morphology contributes to high surface roughness. The inset macrographs in the upper-right corner clearly show the distinct color of the two coatings. This color difference may be attributed to the changes in the electronic structure associated with the incorporation of oxygen vacancies in b-TiO_2_ compared with w-TiO_2_ [29]. The percentage content of the Ti and O elements in the coating surface is shown in Table 1. Notably, the b-TiO_2_ coating exhibited a lower oxygen content than the w-TiO_2_ coating, indicating greater oxygen deficiency consistent with a higher concentration of oxygen vacancies.

The XRD patterns of the b-TiO_2_ and w-TiO_2_ powders are illustrated in Figure 1b. Both powders exhibited the characteristic diffraction peaks of rutile TiO_2_. Specifically, the peaks at 2θ = 27.4°, 36.0°, 41.1° and 54.2° were indexed to the (110), (101), (111) and (211) planes of rutile TiO_2_ (PDF#98-000-0375), respectively. Figure 1c presents the XRD patterns of the corresponding b-TiO_2_ and w-TiO_2_ coatings. No appreciable differences in phase composition were observed between the powders and their respective coatings. However, compared with the TiO_2_ powders, the plasma-sprayed TiO_2_ coatings exhibited a significantly enhanced diffraction intensity of the (101) plane, indicating preferential orientation along this plane. This preferred orientation may result from the rapid melting of TiO_2_ particles in the plasma flame, followed by ultra-rapid solidification when the molten droplets impacted the cold substrate. Under these rapid quenching conditions, the TiO_2_ crystallites may not have sufficient time to nucleate and grow in random orientations. Instead, they preferentially grow along crystallographic directions associated with lower surface energy and more favorable growth kinetics, thereby producing the pronounced (101) texture [30].

### 3.2. Effect of NIR Light Irradiation on Surface Wettability of Coatings

**Figure 2:**
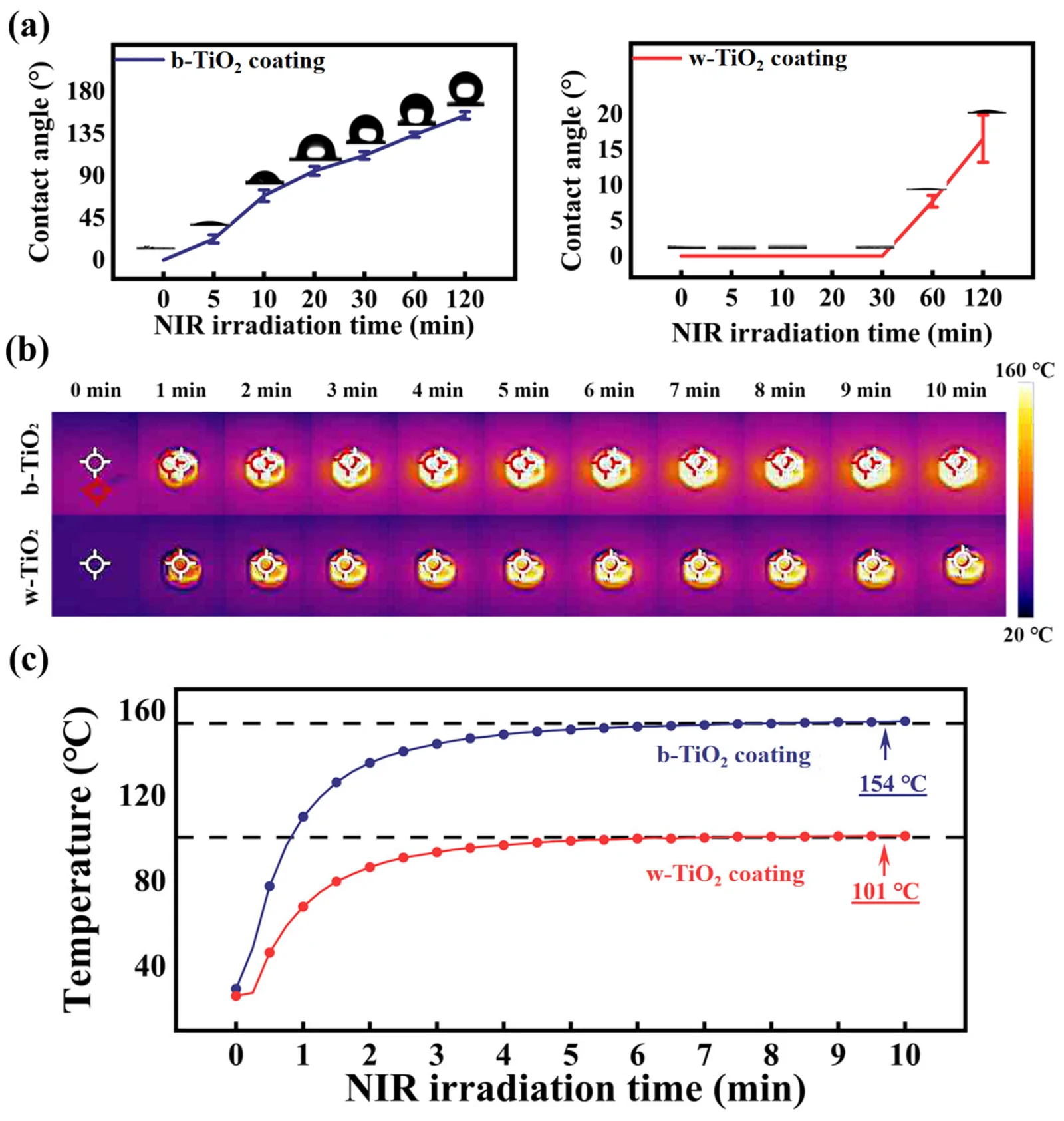
(**a**) Evolution of surface wettability of b-TiO_2_ and w-TiO_2_ coatings under 808 nm NIR irradiation. (**b**) Infrared thermal images of b-TiO_2_ and w-TiO_2_ coatings after NIR light irradiation in air. (**c**) Surface temperature profiles of both coatings as function of NIR irradiation time.

To systematically investigate the effect of light irradiation on the wettability of TiO_2_ coatings, the b-TiO_2_ and w-TiO_2_ coatings were exposed to 808 nm NIR light for 5, 10, 30, 60, and 120 min. The resulting changes in the surface water contact angle are shown in Figure 2a. The water contact angle of the b-TiO_2_ coating gradually increased with irradiation time, reaching 134.0 ± 2.7° after 60 min, indicative of a hydrophobic state, and 154.4 ± 4.0° after 120 min, corresponding to a superhydrophobic state. Thus, NIR irradiation transformed the b-TiO_2_ coating from a superhydrophilic surface, with an initial contact angle of 0°, into a superhydrophobic surface. Moreover, the NIR-induced superhydrophobic state remained stable for more than 200 h. In contrast, NIR irradiation had a negligible effect on the wettability of the w-TiO_2_ coating, whose water contact angle reached only 16.6 ± 3.3° after 120 min under the same irradiation conditions.

The photothermal properties of the coatings were evaluated by monitoring their temperature changes under 808 nm NIR laser irradiation in a dry environment (air). As shown in Figure 2b, the real-time infrared thermal images revealed a pronounced temperature increase, with both coatings exhibiting nearly uniform surface temperature distributions. The corresponding temperature–time profiles (Figure 2c) showed that the surface temperature of the b-TiO_2_ coating rapidly increased to approximately 154 °C within 10 min, whereas that of the w-TiO_2_ coating reached only approximately 101 °C under the same conditions. These results demonstrated that the b-TiO_2_ coating possessed a superior photothermal conversion capability.

The enhanced photothermal response of b-TiO_2_ can be attributed to its altered electronic structure. Conventional w-TiO_2_ is a wide-bandgap semiconductor with a bandgap of approximately 3.0–3.2 eV; therefore, it primarily absorbs UV light and exhibits limited absorption in the visible and NIR regions. By contrast, b-TiO_2_ has a narrower bandgap of approximately 2.4–2.8 eV, which enables broadband light absorption [31]. Following photoexcitation, the absorbed energy can be converted into heat through carrier thermalization and nonradiative relaxation and recombination processes rather than being emitted as photons [32]. Therefore, the difference in photothermal conversion efficiency between the b-TiO_2_ and w-TiO_2_ coatings may be the primary factor underlying their distinct NIR-induced wettability behaviors.

### 3.3. Effect of NIR Light Irradiation on Chemical Properties of Coating Surfaces

**Figure 3:**
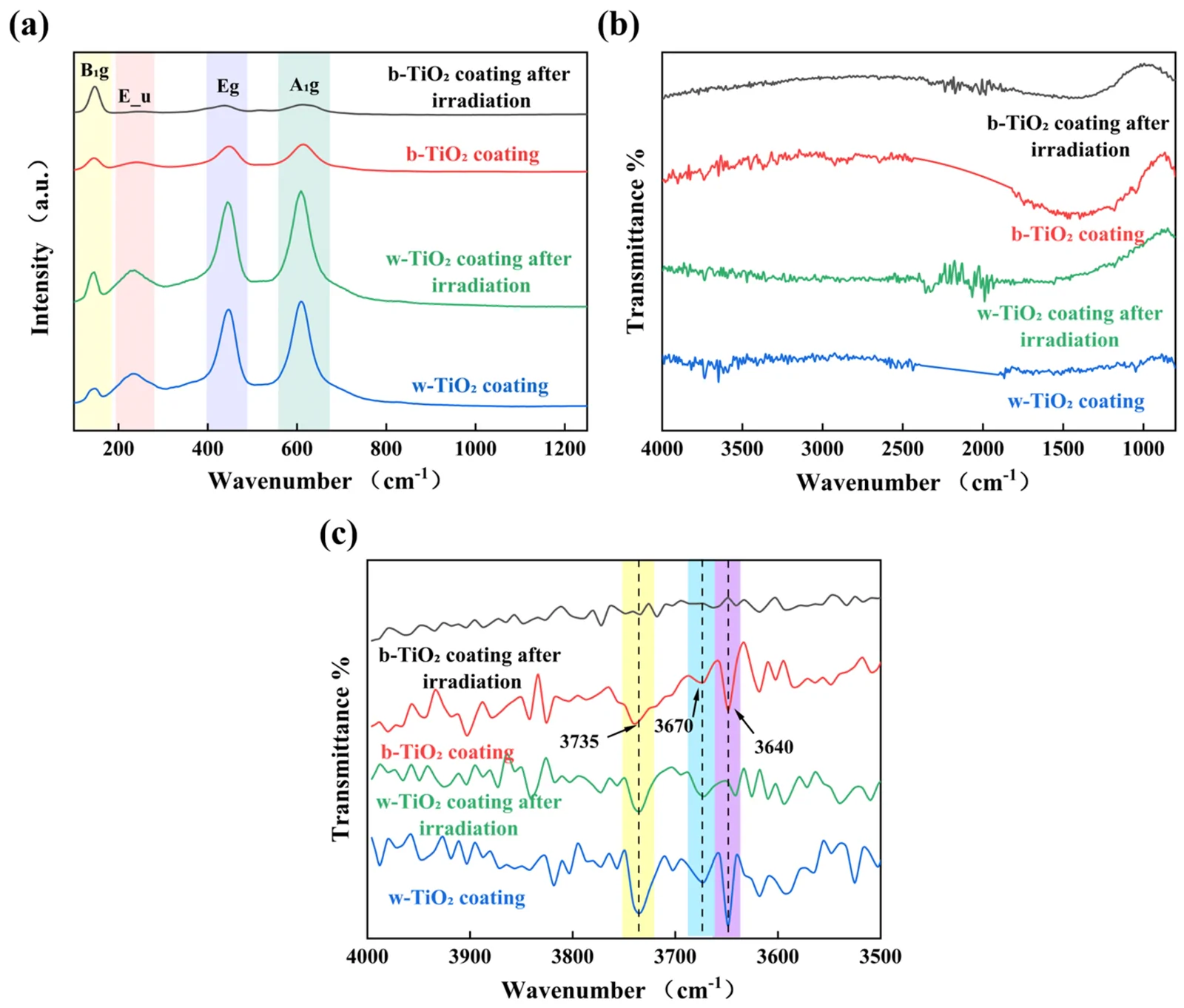
(**a**) Raman spectra, (**b**) full-range FTIR spectra, and (**c**) enlarged FTIR spectra over the selected wavenumber range of b-TiO_2_ and w-TiO_2_ coatings before and after NIR irradiation.

To reveal the mechanism underlying the NIR-induced evolution of surface wettability, changes in the surface chemical properties of b-TiO_2_ and w-TiO_2_ coatings prior to and after NIR irradiation were investigated. Figure 3a shows the Raman spectra of both coatings. The samples exhibited the characteristic Raman bands of rutile TiO_2_ at 143, 256, 445, and 610 cm^−1^, corresponding to the B_1_g, Raman-forbidden/infrared-active, doubly degenerate Eg, and fully symmetric A_1_g vibration modes, respectively [33]. Before irradiation, the w-TiO_2_ coating exhibited sharp, intense Raman bands, whereas those of the b-TiO_2_ coating were substantially broadened and weakened, indicating a pronounced decrease in crystalline order [29].

NIR irradiation produced no appreciable changes in the Raman spectra of either coating. Moreover, no characteristic bands attributable to peroxide species at approximately 860 and 955 cm^−1^ or superoxide species at approximately 1165 cm^−1^ were detected [34]. These findings suggest that the wettability transition was unlikely to be primarily mediated by adsorbed peroxide or superoxide species.

Raman spectroscopy primarily provides information on crystal structure and selected vibrational modes, such as those associated with O–O bonds, but is less suitable for directly characterizing changes in polar surface species, including hydroxyl groups. Thus, Fourier transform infrared spectroscopy (FTIR) was employed to further analyze the surface functional groups of the TiO_2_ coatings (Figure 3b). Surface hydroxyl (OH) groups reduce coating hydrophobicity because TiO_2_ readily interacts with ambient water molecules to form hydrophilic OH groups. Compared with the as-prepared samples, both coatings exhibited decreased intensities of the absorption bands at 3645, 3670, and 3735 cm^−1^ after NIR irradiation (Figure 3c). These bands are assigned to the vibrational modes of surface OH groups [35,36]. The attenuation was substantially more pronounced for the b-TiO_2_ coating, in which the corresponding bands nearly disappeared. Previous studies have demonstrated that the depletion of surface hydroxyl groups contributed to the hydrophobicity of black TiO_2_ surfaces [37,38]. These results indicated that NIR-induced photothermal heating reduced surface hydroxyl density. Owing to its superior photothermal conversion capability, the b-TiO_2_ coating reached a higher surface temperature, facilitating the more extensive removal of surface hydroxyl groups and thereby promoting the transition from superhydrophilicity to superhydrophobicity.

**Figure 4:**
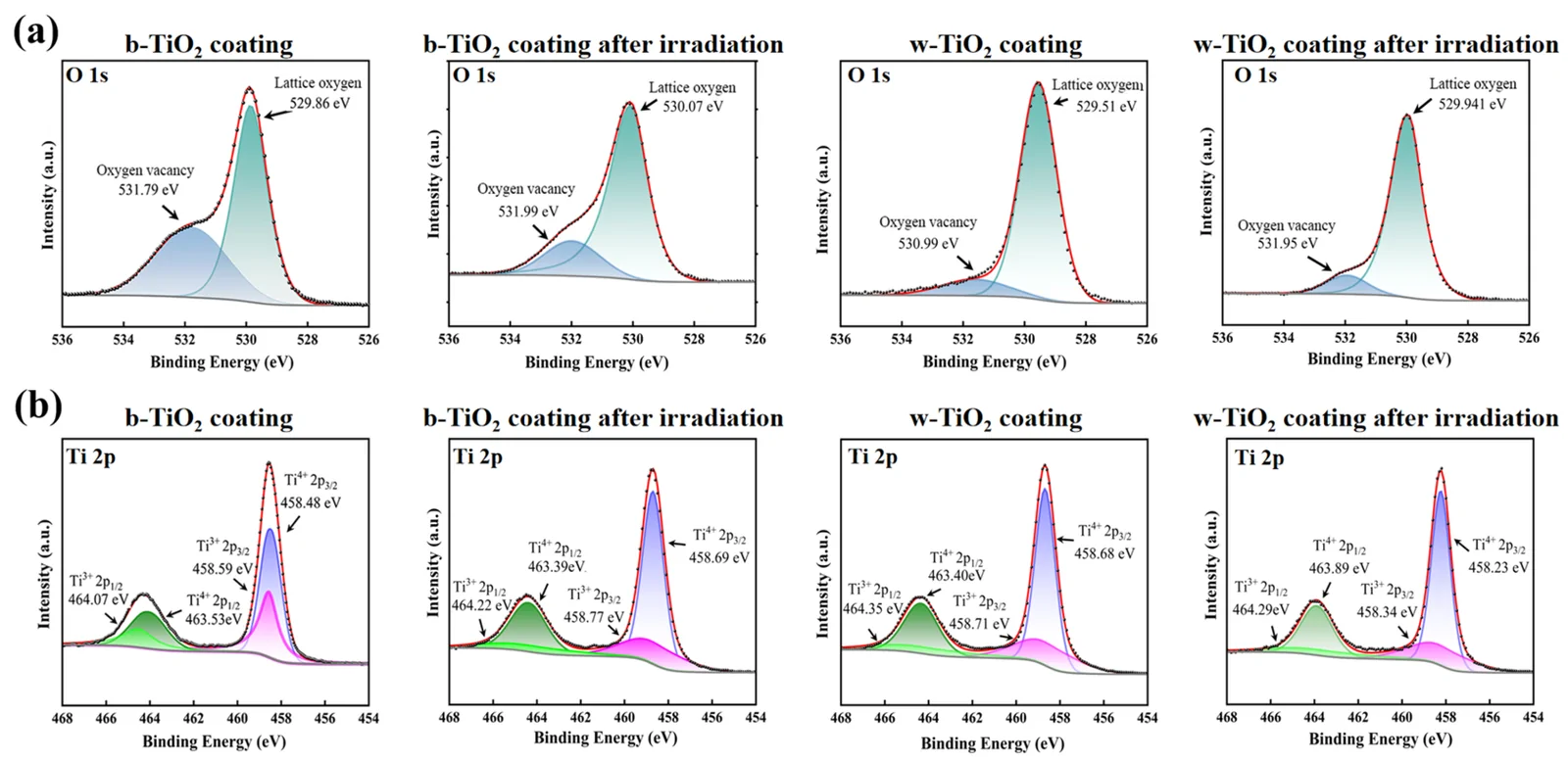
XPS spectra with peak fitting analysis for (**a**) O 1s and (**b**) Ti 2p spectra of b-TiO_2_ and w-TiO_2_ coatings before and after NIR irradiation.

As shown in Figure 4a, each O1s spectrum was deconvoluted into two characteristic peaks. The peak at 529.8 ± 0.6 eV corresponded to lattice oxygen in TiO_2_, while the peak at approximately 531.47 ± 0.5 eV was attributed to oxygen-defect-related species Compared with the w-TiO_2_ coating, the lattice oxygen peak of the b-TiO_2_ coating shifted toward a higher binding energy, likely because oxygen vacancies reduced the local electron density around the remaining oxygen atoms [39]. Following NIR irradiation, the intensity of the oxygen-defect-related peak decreased in both coatings. Quantitative analysis revealed that its relative content decreased from 36.25% to 25.46% in b-TiO_2_ and from 14.76% to 11.57% in w-TiO_2_.

The Ti 2p spectra exhibited the characteristic spin–orbit-split Ti 2p_3/2_ and Ti 2p_1/2_ components. The peaks at 458.5 ± 0.5 and 463.6 ± 0.6 eV were assigned to Ti^4+^, whereas the two weaker shoulder components at 458.6 ± 0.4 and 464.2 ± 0.2 eV were attributed to Ti^3+^ species [40]. The deconvolution of the Ti 2p spectra revealed that Ti^3+^ was nearly undetectable in w-TiO_2_, in which Ti predominantly existed as Ti^4+^. By contrast, b-TiO_2_ contained abundant Ti^3+^ defects and exhibited a shift in its characteristic peaks toward lower binding energies. Following NIR irradiation, the Ti^3+^/Ti^4+^ ratio in b-TiO_2_ decreased from 39.04% to 29.14%, consistent with the observed decrease in oxygen vacancy content.

FTIR and XPS analyses indicate that the photothermally induced surface temperature is a key factor governing the wettability of TiO_2_ coatings. Under 808 nm NIR irradiation, the surface temperature of the b-TiO_2_ coating increased to approximately 154 °C. At this temperature, photothermal heating promoted the desorption of surface-bound water and the depletion of hydroxyl groups, while oxygen adsorption and defect passivation reduced the concentrations of surface oxygen vacancies and Ti^3+^ species. The concurrent decrease in these hydrophilic surface sites substantially enhanced surface hydrophobicity, driving the b-TiO_2_ coating from a superhydrophilic to a superhydrophobic state. By contrast, the w-TiO_2_ coating reached only approximately 101 °C, which was insufficient to remove surface hydroxyl groups to the same extent and therefore produced no pronounced hydrophobic transition. As previously reported [41], atmospheric oxygen can adsorb onto the coating surface and passivate oxygen-vacancy-related defect sites, thereby altering surface chemistry and wettability. Collectively, these findings suggest that the NIR-induced wettability transition of the b-TiO_2_ coating primarily originates from the photothermal desorption of adsorbed water, depletion of surface hydroxyl groups, and passivation of oxygen-deficient sites. Under the conditions examined in this study, the elevated surface temperature of approximately 154 °C effectively promotes the thermal desorption of adsorbed water molecules and surface hydroxyl groups from the coating. The removal of these polar groups lowers the surface free energy and weakens hydrogen bonding between the coating surface and incident water droplets, which consequently increases the water contact angle and renders the surface superhydrophobic.

### 3.4. In Vitro Anti-Adhesion Activity of b-TiO_2_ Coatings with Different Wettability States

To investigate the effect of TiO_2_ surface wettability on bacterial adhesion, b-TiO_2_ coatings with three distinct wettability states were prepared by varying the duration of NIR irradiation. The as-sprayed b-TiO_2_ coating exhibited a water contact angle of 0°, indicating a hydrophilic surface. After 0.3 h of NIR irradiation, the water contact angle increased to 87.9 ± 2.16°, producing an intermediate-wettability surface. Following 2 h of irradiation, the coating exhibited a hydrophobic surface.

**Figure 5:**
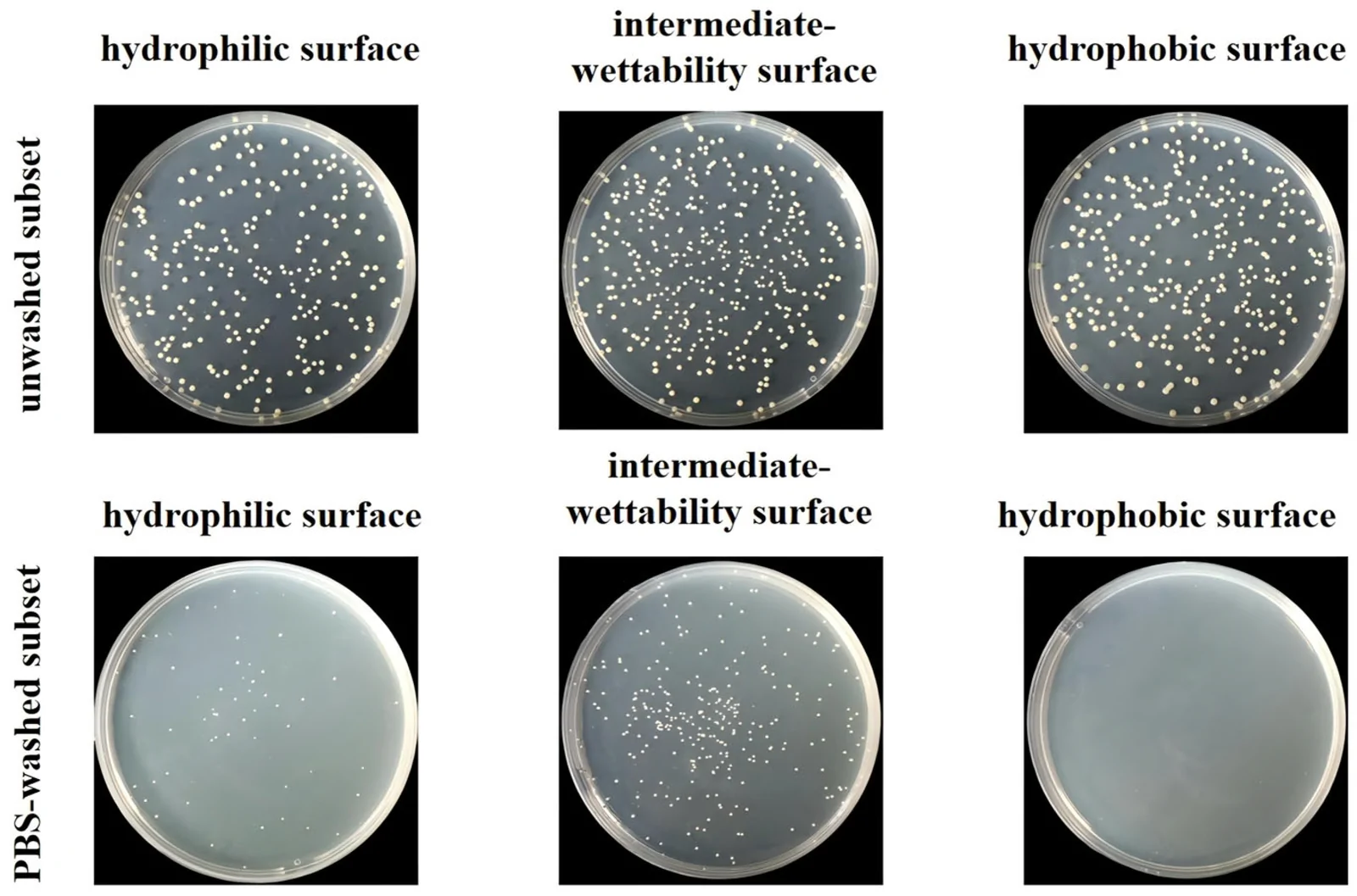
Representative photographs of *S. aureus* colonies recovered from the surface of the hydrophilic, intermediate-wettability, and hydrophobic b-TiO_2_ surfaces.

The in vitro anti-adhesion activity of the coatings against *S. aureus* was evaluated via the plate-counting method (Figure 5). After bacterial incubation on the coated samples for 24 h, the samples were divided into two subsets. Unwashed subset: The coated sample was transferred directly to PBS and ultrasonicated to detach all adhered bacteria. PBS-washed subset: The coated sample was gently rinsed with PBS to remove loosely attached bacteria, then transferred to PBS and ultrasonicated identically. For the unwashed subset, comparable dense bacterial colonies were observed on hydrophilic, intermediate-wettability and hydrophobic surfaces. In contrast, in the PBS-washed subset, all three coatings showed fewer bacterial colonies compared with their unwashed counterparts. Notably, the hydrophobic coating exhibited the strongest anti-adhesion effect, with almost no viable bacterial colonies detected. This indicated that most bacteria on the hydrophobic coating were weakly adhered and readily displaced by PBS washing. The anti-adhesion efficiencies of the intermediate-wettable, hydrophilic, and hydrophobic coatings were 45.91%, 70.13%, and 98.61%, respectively.

Previous studies have demonstrated that surfaces with different wettability states inhibit bacterial adhesion through distinct mechanisms. In particular, highly hydrophobic surfaces generally exhibit strong anti-adhesive properties [42]. This behavior is primarily attributed to the formation of a trapped air plastron at the coating–liquid interface, which maintains a Cassie–Baxter wetting state and substantially reduces the effective solid–liquid contact area available for bacterial adhesion. The trapped air may also buffer interfacial adhesion forces, making it difficult for bacteria to sense the underlying substrate and establish stable attachment [43,44]. Moreover, the extensive air–liquid interface provides few effective anchoring sites, while the limited remaining solid–liquid contact regions are insufficient to support firm bacterial adhesion. Consequently, the interactions between bacteria and the substrate are weakened, reducing both initial bacterial attachment and interfacial adhesion strength and thereby imparting excellent resistance to bacterial adhesion [45,46].

### 3.5. Macrophage Polarization on the b-TiO_2_ Coatings with Different Wettability States

Three b-TiO_2_ coatings with distinct wettability states—hydrophilic, intermediate wettability, and hydrophobic—were used to investigate the effects of surface wettability on macrophage polarization. Flow cytometry was performed to assess the expression of CCR7 and CD206, commonly used as markers of M1- and M2-like macrophage phenotypes, respectively.

**Figure 6:**
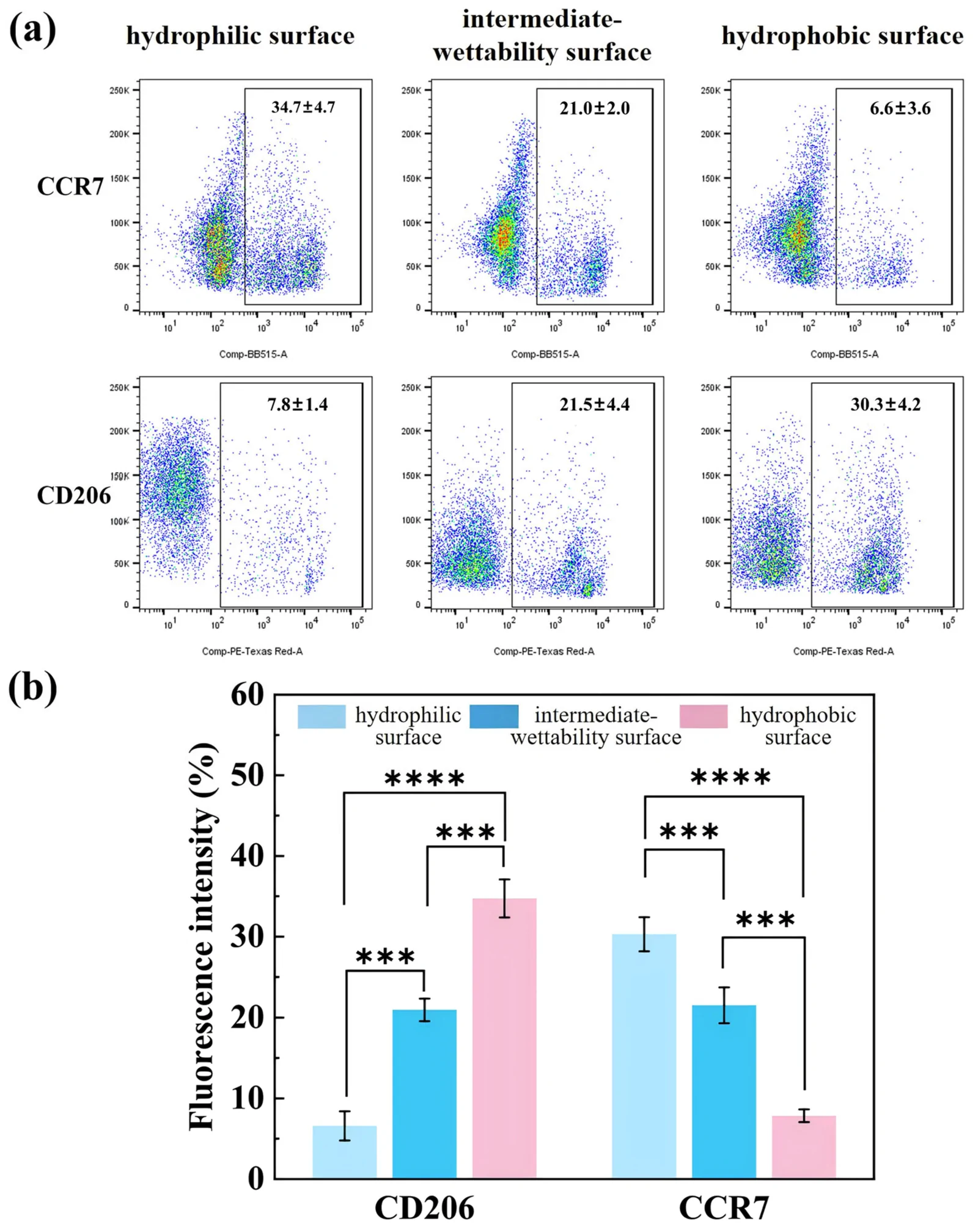
(**a**) Representative flow cytometry plots of RAW264.7 macrophages stained for CCR7 and CD206. (**b**) Quantitative analysis of CCR7 and CD206 expression ratio. The degree of significance was indicated by *** *p* < 0.001 or **** *p* < 0.0001.

As shown in Figure 6a, with increasing surface hydrophobicity, the proportion of CCR7-positive cells progressively decreased from 34.7 ± 4.7% to 21.0 ± 2.0% and then to 6.59 ± 3.6%. Conversely, the proportion of CD206-positive cells increased from 7.8 ± 1.4% to 21.5 ± 4.4% and reached 30.3 ± 4.2% on the hydrophobic coating. Statistical analysis further revealed significant differences among the three groups (Figure 6b). These findings indicate that increasing the surface hydrophobicity of the b-TiO_2_ coating suppressed M1-like macrophage polarization while promoting an M2-like phenotype.

**Figure 7:**
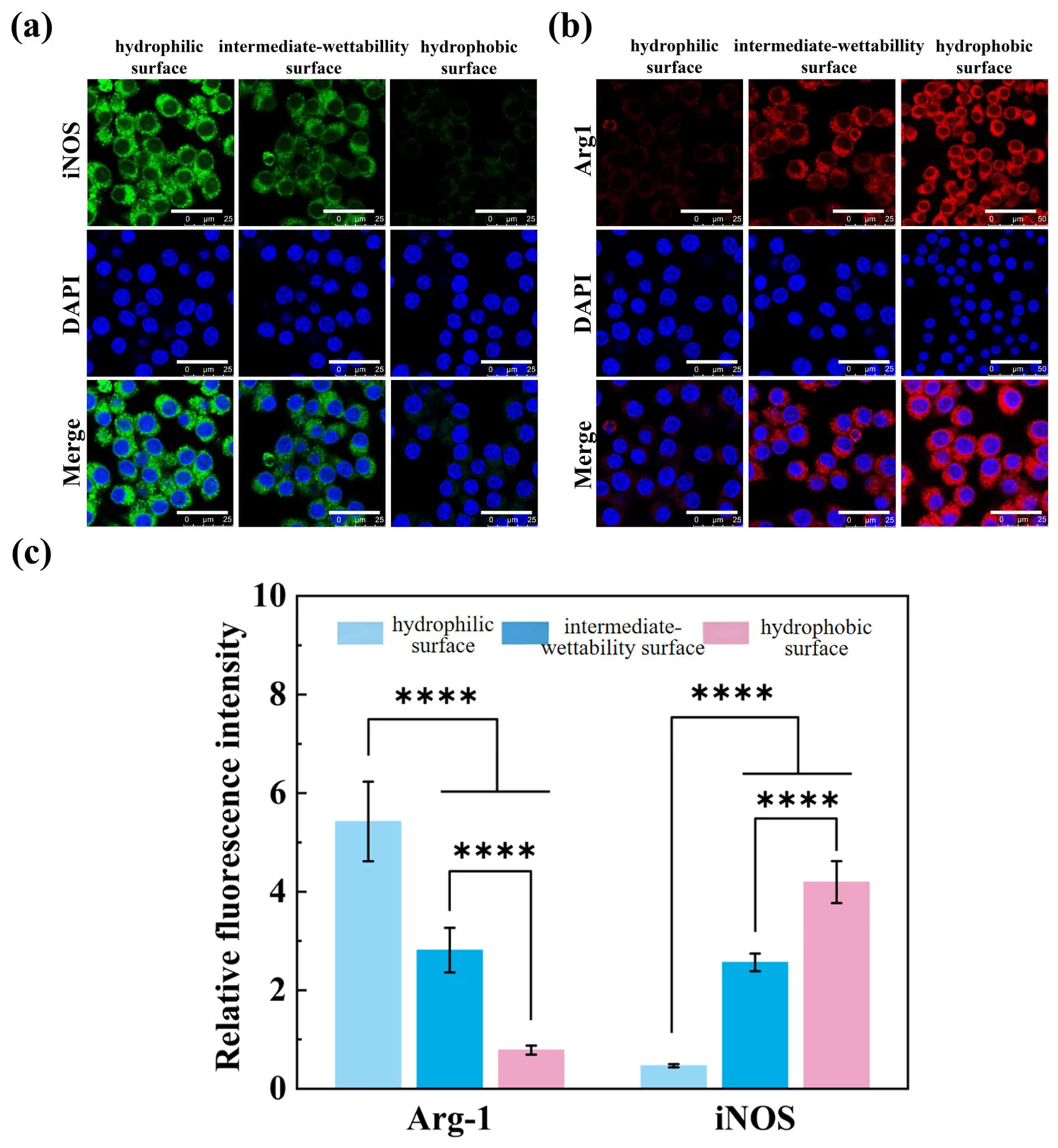
Representative immunofluorescence images of macrophages cultured on the coating surfaces and stained for (**a**) iNOS (green) and (**b**) Arg-1 (red). Scale bar: 25 μm. (**c**) A quantitative analysis of iNOS and Arg-1 expression. The degree of significance was indicated by **** *p* < 0.0001.

During acute inflammation, M1-like macrophages produce nitric oxide (NO) through inducible nitric oxide synthase (iNOS). In contrast, M2-like macrophages contribute to tissue repair and wound healing through arginase-1 (Arg-1), which converts L-arginine into L-ornithine. Therefore, macrophage polarization on the different coating surfaces was further assessed by the immunofluorescence staining of iNOS and Arg-1 in RAW264.7 macrophages. As shown in Figure 7, the intermediate-wettability and hydrophobic surfaces exhibited lower iNOS fluorescence intensities than the hydrophilic surface, with the hydrophobic surface showing the lowest intensity. This trend was consistent with the flow cytometry results, suggesting that increasing surface hydrophobicity suppressed M1-like macrophage polarization. Conversely, Arg-1 fluorescence intensity was higher on the intermediate-wettability and hydrophobic surfaces than on the hydrophilic surface, with the highest intensity observed in the hydrophobic group. Collectively, these findings indicated that the hydrophobic b-TiO_2_ surface suppressed M1-like polarization while promoting an M2-like macrophage phenotype.

Previous studies have shown that surface wettability can influence macrophage polarization [47,48]. Del Carmen Morán et al. reported that the low surface energy of hydrophobic materials altered the adsorption and conformation of adhesion-related proteins, such as fibronectin and vitronectin. These changes may inhibit focal adhesion formation, reduce macrophage attachment, suppress NF-κB-mediated inflammatory signaling, and promote M2-like polarization [49]. Rostam et al. further demonstrated that hydrophobic interfaces modified the composition and conformation of adsorbed proteins, weakened integrin signaling, and suppressed M1-associated pathways involving STAT1 and IRF5, thereby promoting an M2-like phenotype [50]. However, the influence of hydrophobicity on macrophage polarization is not universal. Some hydrophobic surfaces have been reported to induce pro-inflammatory M1-like polarization, suggesting that wettability acts together with surface roughness and chemical composition. Hotchkiss et al. found that smooth hydrophobic surfaces tended to promote M1-like polarization, whereas rough hydrophobic surfaces were more likely to favor an M2-like phenotype [51]. Surface oxygen content may also influence macrophage behavior by altering the composition, orientation, and conformation of the adsorbed protein layer [50].

Taken together, these findings indicate that implant surface wettability is an important physicochemical factor regulating macrophage behavior, although its effects depend on the accompanying surface topography and chemistry. In the present study, the NIR-induced hydrophobic b-TiO_2_ coating reduced M1-like polarization and promoted an anti-inflammatory M2-like phenotype, which may provide a favorable immune microenvironment for subsequent osteoblast activity.

### 3.6. Effects of Surface Wettability States of b-TiO_2_ Coatings on Osteoblast Functions

**Figure 8:**
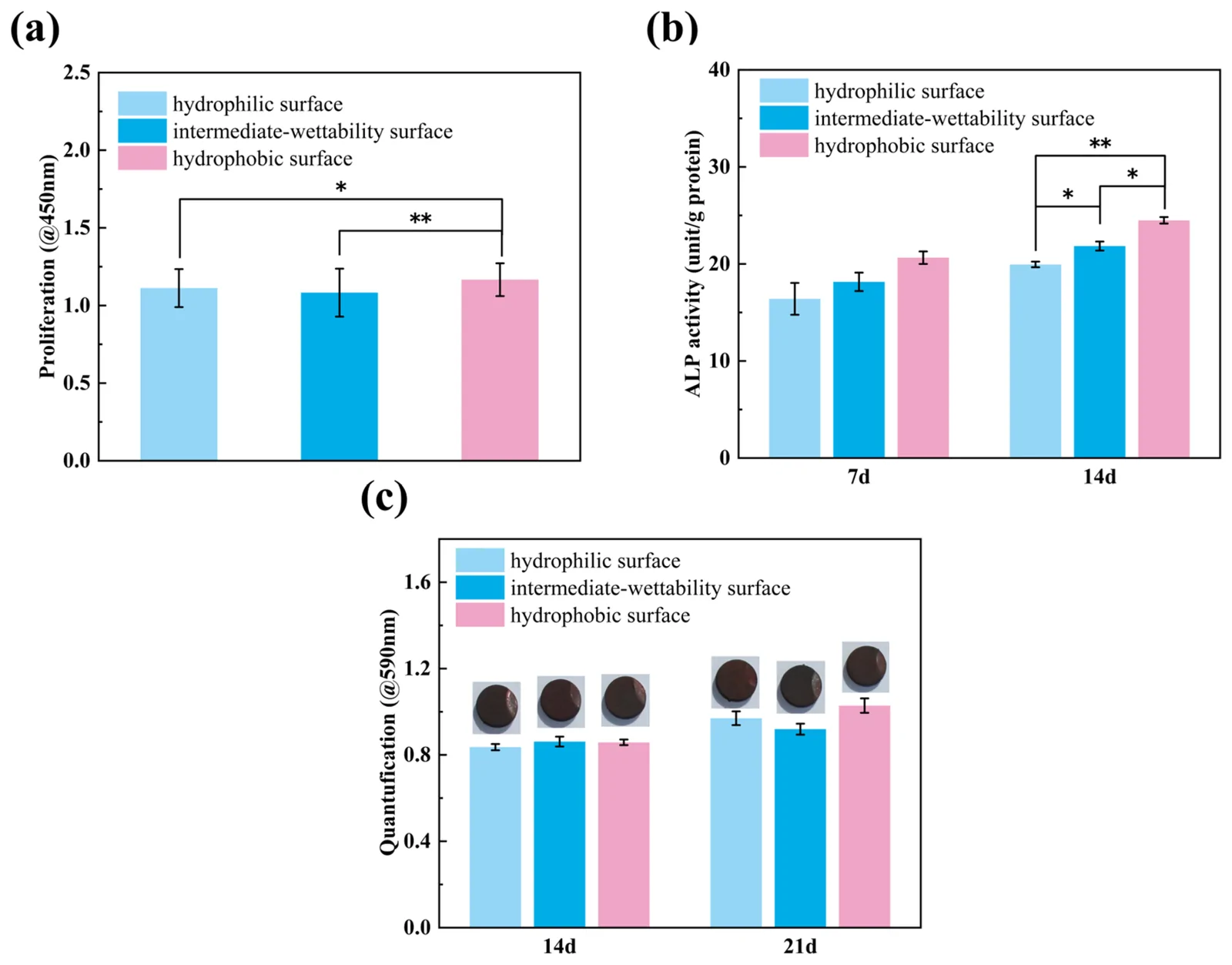
(**a**) Viability of MC3T3-E1 cells on hydrophilic, intermediate-wettability, and hydrophobic b-TiO_2_ surfaces. (**b**) ALP activity of cells cultured on coatings for 7 and 14 days. (**c**) Calcium deposition by cells cultured on coatings for 14 and 21 days. The degree of significance was indicated by * *p* < 0.05, ** *p* < 0.01.

MC3T3-E1 cells were cultured on hydrophilic, intermediate-wettability, and hydrophobic b-TiO_2_ surfaces for 7 days, and cell proliferation was evaluated (Figure 8a). Although statistically significant differences were observed among the three groups, the overall differences were minor. ALP is an early marker of osteogenic differentiation [52,53]. As shown in Figure 8b, there was no statistically significant difference in ALP activity among the three groups on day 7 of osteogenic induction. On day 14, the hydrophobic group possessed higher ALP activity than the other two groups, which was 22.81% and 12.13% higher than that of the hydrophilic group and intermediate-wettability group, respectively.

Calcium deposition is an indicator of extracellular matrix mineralization and was evaluated by alizarin red S (ARS) staining [53]. A quantitative analysis of ARS staining showed that calcium deposition increased with culture time (Figure 8c). The staining results demonstrated that calcium deposition continuously increased in all test groups with the extension of cell culture time. There was barely any distinction between groups on day 14. When the culture reached day 21, the coating with moderate surface wettability generated the least calcium sediment. To be precise, the calcium mineral deposition of the hydrophilic coating and hydrophobic coating was 6% and 12% higher than that of the moderately wettable coating, respectively.

Previous studies have widely demonstrated that hydrophilic surfaces are more conducive to osteogenic proliferation, differentiation and mineralization [54,55,56]. Nevertheless, the present b-TiO_2_ coatings yielded different results. The hydrophobic b-TiO_2_ coating fabricated in this study does not exhibit deteriorated osteogenic properties; instead, it delivers slightly superior osteogenic activity compared with hydrophilic counterparts. This unique phenomenon originates from the biological inertness of b-TiO_2_. For such coatings, surface roughness may exert a far more dominant regulatory effect on osteogenic behavior than surface wettability. This dominant positive effect offsets the inherent disadvantage of hydrophobic surfaces in osteogenesis, thereby endowing the coating with favorable osteogenic capacity [57,58,59].

## 4. Conclusions

In this study, b-TiO_2_ coatings with NIR-responsive wettability were fabricated directly on a Ti substrate using a one-step APS process. After 2 h of NIR irradiation, the b-TiO_2_ coating transitioned from a superhydrophilic state to a stable superhydrophobic state, with a contact angle of 154.4 ± 4.0°. The robust hydrophobicity of the NIR-modified b-TiO_2_ coating can be stably maintained for over 200 h, which bears great clinical significance for orthopedic implants given that the 0–7-day post-implantation period represents a high-risk window for bacterial colonization and postoperative infection. In contrast, the conventional w-TiO_2_ coating remained hydrophilic under identical irradiation conditions. This wettability transition was primarily attributed to NIR-induced photothermal heating, which promoted the desorption of adsorbed water and surface hydroxyl groups and the passivation of oxygen-deficient sites, thereby reducing the surface free energy. By varying the duration of NIR irradiation, b-TiO_2_ coatings with three distinct surface wettability states were produced—hydrophilic, intermediate wettable, and hydrophobic. Among them, the hydrophobic b-TiO_2_ coating effectively suppressed *S. aureus* adhesion, achieving an antibacterial adhesion efficiency of 98.61%. Compared with the hydrophilic and intermediate-wettable counterparts, the hydrophobic b-TiO_2_ coating preferentially induced macrophage polarization toward the anti-inflammatory M2 phenotype and delivered slightly superior ALP activities of osteoblasts. Overall, the NIR-induced regulation of plasma-sprayed b-TiO_2_ coatings provides a potential strategy for developing orthopedic implant surfaces with combined antibacterial and immunomodulatory properties.

## Figures and Tables

**Table 1:** The percentage of the content of each element in b-TiO_2_ and w-TiO_2_ coatings.

Coating	O	Ti
b-TiO_2_	60.5%	39.5%
w-TiO_2_	68.4%	31.6%

## Data Availability

The original contributions presented in this study are included in the article. Further inquiries can be directed to the corresponding authors.
